# Enhancing Lung Cancer Survival Prediction: 3D CNN Analysis of CT Images Using Novel GTV1-SliceNum Feature and PEN-BCE Loss Function

**DOI:** 10.3390/diagnostics14121309

**Published:** 2024-06-20

**Authors:** Muhammed Oguz Tas, Hasan Serhan Yavuz

**Affiliations:** Electrical and Electronics Engineering Department, Eskisehir Osmangazi University, Eskisehir 26480, Turkey; hsyavuz@ogu.edu.tr

**Keywords:** lung cancer, survival classification, 3D CNN, *PEN-BCE* loss, *GTV1-SliceNum*, medical image analysis

## Abstract

Lung cancer is a prevalent malignancy associated with a high mortality rate, with a 5-year relative survival rate of 23%. Traditional survival analysis methods, reliant on clinician judgment, may lack accuracy due to their subjective nature. Consequently, there is growing interest in leveraging AI-based systems for survival analysis using clinical data and medical imaging. The purpose of this study is to improve survival classification for lung cancer patients by utilizing a 3D-CNN architecture (ResNet-34) applied to CT images from the *NSCLC-Radiomics* dataset. Through comprehensive ablation studies, we evaluate the effectiveness of different features and methodologies in classification performance. Key contributions include the introduction of a novel feature (*GTV1-SliceNum*), the proposal of a novel loss function (*PEN-BCE*) accounting for false negatives and false positives, and the showcasing of their efficacy in classification. Experimental work demonstrates results surpassing those of the existing literature, achieving a classification accuracy of 0.7434 and an ROC-AUC of 0.7768. The conclusions of this research indicate that the AI-driven approach significantly improves survival prediction for lung cancer patients, highlighting its potential for enhancing personalized treatment strategies and prognostic modeling.

## 1. Introduction

Lung cancer is very common worldwide and is one of the cancer types with the highest mortality rate. The symptoms of lung cancer usually appear in the later stages of the disease and tend to spread (metastasis) to other organs/tissues, causing the disease to be fatal. Estimated new cases of lung cancer are second only to prostate cancer in men and breast cancer in women, and estimated mortality rates from lung cancer are the highest in both men and women at 21% [1].

With survival analysis, which allows us to estimate the time until an event occurs [2], the time of disease recurrence or death of the patient can be predicted. This prediction is crucial to shaping the treatment processes of cancer patients, as it helps clinicians make informed decisions about treatment plans and monitoring strategies. For example, patients with a poor prognosis can be monitored more closely and benefit from more aggressive treatment and advanced care planning [3], while standard treatment protocols with regular monitoring can be applied to patients with a better prognosis. Survival analysis also allows us to understand the course and consequences of the disease by evaluating the prognosis of different types of cancer and examining the survival rates of cancer patients within a certain period from the diagnosis and the factors affecting these rates.

Although the survival rate of lung cancer varies depending on the stage of diagnosis, the type of cancer, and the general health status of the patient, Siegel et al. expressed the 5-year relative survival rate for lung and bronchus cancer between 2012 and 2018 as 23% in the United States [1]. When deciding on the survival rate, clinicians take into consideration factors such as the patient’s age, gender, smoking status, lifestyle, genetic structure, stage of the disease, and the tumor location and its tendency to spread. However, it may be complicated for a clinician to make a survival prediction by considering a vast amount of different information, bringing all this information together simultaneously, and drawing meaningful conclusions from it. Additionally, analyses that are contingent upon the assessment of clinical physicians may yield inaccurate results due to their reliance on the physician’s subjective interpretation, observation, intuition, and knowledge.

Due to advancements in technology, it is recognized that deep learning and machine learning algorithms have the capability to efficiently analyze vast datasets, swiftly process intricate information, and uncover underlying patterns by automatically extracting features from the data. Therefore, artificial intelligence-based survival analyses using clinical data and/or images from imaging devices such as Magnetic Resonance (MR), Computed Tomography (CT), and Positron Emission Tomography (PET) can produce more reliable and more accurate results.

Although survival analysis can leverage a variety of data sources, including clinical data, radiomics data, genetic information, and histopathological images, as well as CT, MR, and PET images, employing diverse methodologies, the lifetime estimation problem requires diversified approaches due to censored observations. A large number of studies of lung cancer patients have explored survival analysis, employing a spectrum of approaches, from conventional statistical methods [4,5] to machine learning models [6,7]. With advancing technology, it is evident that deep learning algorithms can efficiently process vast datasets, automatically extracting features and handling complex data with speed and effectiveness. Mukherjee et al. proposed a shallow network called LungNet, to predict the survival of different Non-Small Cell Lung Cancer (NSCLC) patients from four medical centers and presented this network as two different versions according to the input image (one version takes only CT images as input, the other creates input data by combining CT images with clinical data such as age, gender, histology, and cancer stage). In addition, LungNet was used to classify nodules as benign or malignant [8]. Zhu et al. presented a solution that integrates genetic data and pathological images for the first time for lung cancer survival. They extracted various features (geometry, texture, and holistic) by segmenting cells from pathological images, and then performed dimension reduction and survival analysis [9]. In another study, Zhu et al. developed a deep convolutional neural network model, called DeepConvSurv, for survival analysis using pathological images, and stated that, in comprehensive experiments on National Lung Screening Trial (NLST) cancer data, the proposed model provided significant improvement over state-of-the-art methods [2]. Again, Zhu et al. proposed a method called Whole Slide Images Survival Analysis (WSISA), which enables predictions to be made at the cluster level with the DeepConvSurv model by first extracting parts from the image and then grouping these partial images into different clusters [10]. Dao et al. proposed a transformer-based Multi-scale Aggregation-based Parallel Transformer Network (MAPTransNet) that segmented tumor cells and normal tissues in 3D PET/CT images, then proposed a Multimodality Survival Network (MSNet) for survival analysis to estimate the hazard ratio of patients; they used the segmented region of interest (RoI) and clinical data [11]. Haarburger et al. performed survival analysis using radiomics data and CT images together. Using the segmentation images in the database, different radiomic features (18 statistics, 15 shapes, and 73 textures) were extracted, and important features were selected. In order to extract CNN features, the ResNet18 model was used. Then, CNN features and radiomic features were combined and the Cox PHM module was added to the output of the model [12]. Wu et al. proposed a three-phase (multi-modal feature extraction with 3D ResNet for CT images and two hidden layer deep neural networks for clinical data, multi-modal fusion of features with early fusion, and survival analysis) architecture called DeepMMSA that can fully utilize CT images and clinical information to improve the survival prediction accuracy of lung cancer patients [13]. In their subsequent work, Wu et al. proposed a two-tower survival analysis network called Lite-ProSENet, which takes clinical data and CT scans as input. The textural tower is responsible for modeling clinical data, while the visual tower is responsible for extracting features from CT scans. Comprehensive experiments were carried out in the study, and they showed that Lite-ProSENet outperformed the other studies considering the c-index metric [14].

In the literature, survival analysis is widely considered as a survival classification problem for lung cancer. The survival classification problem focuses on individuals classifying whether an event (death) will occur within a certain time interval. The classification problem is generally evaluated as 2-class (1-year, 2-year, 5-year) or 3-class (Class 1: ≤ 6-month, Class 2: 6–24 months, Class 3: ≥ 24-month or Class 1: ≤36-month, Class 2: 36–60 months, Class 3: ≥60-month), using the determined threshold as reference. Using Machine Learning (ML) or Convolutional Neural Network (CNN) models trained with various methods, the success of the models is evaluated with many classification metrics, especially accuracy (ACC) and area under the curve (AUC) metrics. Doppalapudi et al. addressed survival analysis on the lung section of the SEER dataset as a classification and regression problem. In the study, Artificial Neural Network (ANN), Recurrent Neural Network (RNN), Convolutional Neural Network (CNN), Random Forest (RF), Support Vector Machines (SVM), and Naïve Bayes models were compared with different metrics to solve the 3-class survival classification problem, and the authors emphasized that the ANN-based model was more successful than other models, with the best accuracy result [15]. Lai et al. developed a multimodal deep neural network combining gene expression profiles and clinical data to accurately predict the 5-year overall survival of Non-Small Cell Lung Cancer (NSCLC) patients. In the study, survival status was estimated with 15 biomarkers combined with clinical data, and the results were compared with other well-known classifiers (K-Nearest Neighbors (KNN), RF, SVM) [16]. Tang et al. introduced a new capsule network called CapSurv with a new loss function called survival loss to perform survival analysis with whole slide pathological images. In the study, semantic-level features extracted by VGG16 are used to train CapSurv to distinguish distinctive patches from whole slide pathological images. The method was tested as a 1-year survival classification problem in two different datasets and showed that the proposed CapSurv model could improve the prediction performance [17]. Paul et al. utilized transfer learning to extract deep features from CT images of lung cancer patients, then, in order to predict short- and long-term survivors, classifiers were trained [18]. Han et al. proposed a new multi-branch spatiotemporal residual network (MS-ResNet) for disease-specific survival prediction by integrating CT images and clinical data. This model extracts deep features from CT images with an improved residual network. With the feature selection algorithm, the most relevant subset of features is selected from the clinical data. Finally, the features are combined to leverage the two data types. Experiments have shown that it provides better results than other methods in the literature for the short, medium, and long survival classification problems [19]. Wang et al. proposed an unsupervised deep learning method (residual convolutional autoencoder) to take advantage of unlabeled data in survival analysis and observed that deep learning features gave better results than radiomic features in 1-year classification [20]. Parmar et al. compared fourteen feature selections and twelve classification methods on their performance in predicting the overall survival of lung cancer by utilizing the *Lung1* dataset, which is also employed here. In the study, a total of 440 radiomic features were extracted from the patients’ pre-treatment CT images. It has been demonstrated that the feature selection method based on the Wilcoxon test and the Random Forest classification method has the highest prognostic performance and stability [21]. In order to show that the tissue surrounding the tumor is also clinically important, Vial et al. estimated the 2-year survival classification of the annular region by extracting tissue features from the outer part of the tumor. In the study, it was shown that radiomic features obtained from regions located outside but close to the tumor also have prognostic value [22]. Braghetto and Braghetto et al. handled the survival analysis of lung cancer patients as a 2-year cut-off classification problem and compared the performance of radiomics and deep learning-based methods in survival prediction. The study included the CNN module, which provides direct feature extraction from CT images, the radiomic features module, and the module in which the obtained features are subjected to feature selection and dimensionality reduction. Deep learning-based applications gave worse results than radiomic data due to the lack of data in the study, inaccurate reconstruction with Convolutional Auto Encoder (CAE), and poor synthetic data produced with the Generative Adversarial Network (GAN) [23,24].

In Table 1, we provide a comprehensive overview of the literature studies addressing the survival classification problem for lung cancer. Each study is meticulously documented with key details including reference, cancer type, the dataset used, the model employed (highlighting the best-performing model where applicable), classification type, and corresponding performance metrics. This compilation offers insights into the diverse methodologies and performance outcomes achieved in survival classification research.

In this study, our main purpose is to enhance lung cancer survival prediction by using modern Artificial Intelligence (AI) based methodologies. Toward this aim, we conducted analyses for survival classification from CT images of lung cancer patients, using a publicly available lung cancer database, and performed ablation studies to assess the classification success. Along with this, we provided a comprehensive literature analysis for the lung cancer survival analysis. The primary contributions of this study can be summarized as follows:

- Introduction of a novel feature termed *GTV1-SliceNum*, which considers the number of Gross Tumor Volume-1 (GTV-1) tumor-containing slices in patients’ CT scans. Its integration into the clinical data and its impact on classification success are demonstrated.
- Proposal of a new loss function, *Penalized Binary Cross Entropy Loss (PEN-BCE)*, designed to account for false negative (FN) and false positive (FP) values. The effect of this loss function on classification performance is elucidated.
- Surpassing benchmarks established in the existing literature. In this study, performance is evaluated with ACC and AUC metrics. The 2-year survival classification results have been obtained as 74.34% and 77.68%, respectively, both of which exceed the benchmarks established by methods found in the literature.

The remainder of this study is structured as follows: in Section 2, the proposed method is provided; experimental studies and ablation studies are presented in Section 3; findings are analyzed in Section 4, and conclusions are supplied in Section 5.

## 2. Materials and Methods

**Figure 1:**
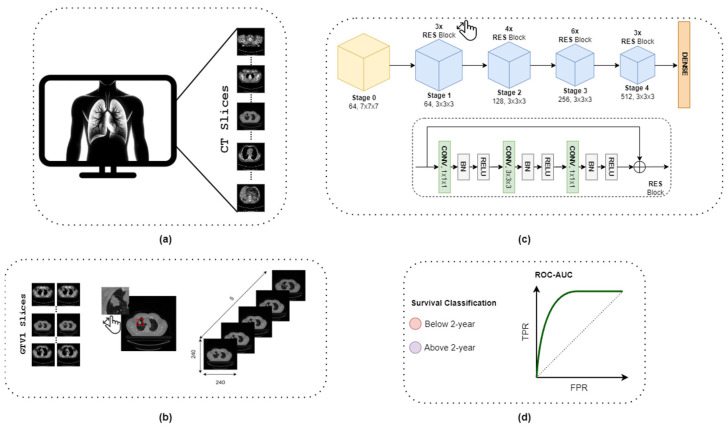
Block diagram of the proposed methodology. (**a**) Original CT slice images in *Lung1* database. (**b**) Pre-processing steps including detection of slices containing GTV-1, finding the slice with the largest tumor area, detection of neighborhood slices of this slice, and preparation of the 5-slice input image in accordance with the CNN model. (**c**) Training 3D ResNet-34 model with BCE and PEN-BCE losses for survival classification. (**d**) The 2-year cut-off survival classification results and evaluation with ROC-AUC performance metric.

Survival analysis is a statistical method used to analyze time-to-event data that is often applied in medical research to study the time until an event of interest occurs, such as death or disease recurrence. In this study, we employed a survival analysis to classify whether individuals would die within a certain time. Patients’ lung CT images were evaluated using a three-dimensional convolutional neural network model to predict the survival time interval. In this context, we used a well-performing 3D CNN architecture which yields outstanding performance on visual object detection in computer vision applications by properly modifying the model to be used for lifetime estimation. The core modifications implemented in the architecture can be succinctly encapsulated in two key alterations. Firstly, the integration of a novel loss function into the model. Traditional computer vision paradigms typically benefit from ample and balanced sample sizes per class during the training phase. However, in the context of the present study, despite leveraging the most extensive labeled dataset available in the domain, namely the *Lung1 dataset*, class sample distributions remain imbalanced. Consequently, a custom loss function has been devised to address this challenge. Secondly, we used an additional feature in clinical observations. These two alterations have resulted in enhanced performance in the prediction of life expectancy. The general block diagram of the method applied in this manuscript is given in Figure 1. 

Further elaboration on the methodology employed in this study will be expounded upon in subsequent sections, following the introduction of data representation and descriptions in the next subsection.

### 2.1. Data Representation and Descriptions

Lung cancer is common worldwide, and the most common lung cancer is Non-Small Cell Lung Cancer (NSCLC), with a rate of 80–85% [25]. Lung cancer can be identified through various methods, including imaging tests, biopsy, sputum cytology, blood tests, or molecular testing. Since our study focuses on predicting survival time from CT images of lung cancer patients, CT imaging data was used as the primary form of data in this study. Among the openly shared datasets available for this purpose, the *NSCLC-Radiomics (Lung1)* dataset emerges as the most suitable for addressing the problem in our study. *NSCLC-Radiomics*, also known as the *Lung1* dataset, is CT imaging data from 422 publicly available NSCLC patients available at The Cancer Imaging Archive (TCIA) [26,27,28]. In this dataset, there is a file with a CSV extension containing the clinical data and three folders containing CT slices, segmentation images, and information about the segmentation images in the folder for each patient. Details of the clinical and CT image data in the dataset are given in the following sections.

#### 2.1.1. Tabular Data (Clinical Information)

Clinical data in the dataset are in a file named *NSCLC Radiomics Lung1.clinical-version3-Oct 2019.csv*. This file includes information about the age of the patients, the T, N, M stages of the cancer, overall stage, histology, gender, survival time, and survival status. A description of the clinical data in the dataset is briefly given in Table 2.

Survival times of patients in the dataset vary between 10 days and 4454 days. In addition, 373 of 422 patients consist of uncensored data, and 49 of them consist of censored data.

#### 2.1.2. Image Data (CT and RTSTRUCT Information)

In the dataset, CT images are kept in folders defined by PatientID in the CSV file for each patient. Within each folder, there are three folders (CT slices, segmentation images, and the Radiotherapy Structure Set (RTSTRUCT) file [29], which contains regions of interest (ROI) information and is used to transfer patient structures and related data between devices in the radiotherapy department).

**Figure 2:**
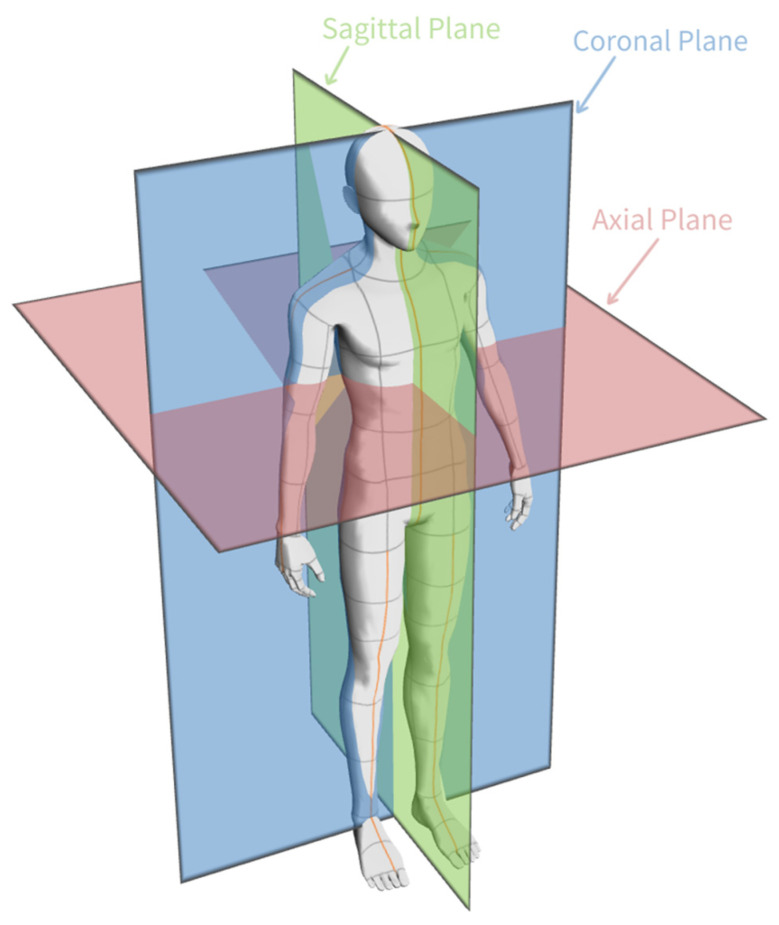
CT images from different cross-sectional areas (coronal, sagittal, axial).

Computed tomography images are 3D images that are composed of many consecutively taken 2D images of a patient. Computed tomography images can be taken from three different cross-sectional areas: axial, sagittal, and coronal. Tomography images of the lung patients used in the study were obtained in the axial plane. An illustration of how coronal, sagittal, and axial planes are obtained from a patient is given in Figure 2.

CT image data in the folders is set in DICOM (Digital Imaging and Communications in Medicine) format, which is a standard protocol for the management and transmission of medical images and related data [30]. DICOM data contains metadata with different names. By using this data, images in DICOM format can be preprocessed and details of the images can be obtained. Within the scope of this study, a web application developed by Innolitcs to enable software developers, researchers, and radiologists to easily navigate the DICOM standard was used to learn the meaning of tag information about DICOM data [31].

CT images of patients in the *Lung1* dataset contain different numbers of slices (75–297). Meta-data specific to the DICOM format described in Table 3 were used to analyze the available slices in this study. The information provided is crucial for the correct preprocessing of the image.

There are segmented images with different labels (GTV, Spinal-Cord, Lung-Left, Lung-Right, Esophagus) for each patient in the dataset. Among these labels, GTV contains location information for the gross tumor volume, Lung-Left for the left lung, Lung-Right for the right lung, Spinal-Cord for the spinal cord region, and Esophagus for the esophagus.

**Figure 3:**
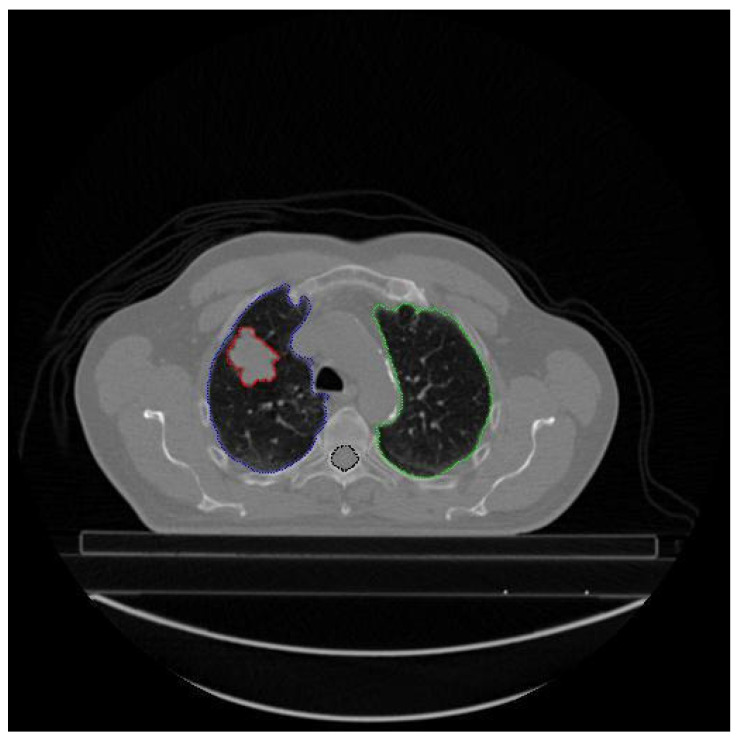
Different types of segmentation data contained in a CT slice (28) of a patient (LUNG1-243).

Each patient’s file may contain different types and numbers of segmented images. The type and number of segmented images are not standard. For example the number of CT images (number of slices) taken from patient LUNG1-001 is 134, and there are a total of 358 segmented images labeled 139 Left-Lung, 134 Right-Lung, 84 Spinal-Cord, and 21 GTV-1, while LUNG1 has 94 slices, LUNG1-243 has a total of 327 segmented images, 113 of which are labeled Left-Lung, 101 Right-Lung, 94 Spinal-Cord, 6 GTV-2, and 13 GTV-1. Additionally, different types and/or numbers of segmented image data may be present in different slices of the same patient. For example, different types of segmented data in CT slice number 28 of patient LUNG1-243 are given in Figure 3.

As seen in Figure 3, segmented data with all different labels may not be present in a slice of a patient. This is because, in the axial CT image, each slice represents a scan of a specific region of the lung. In the example above, blue indicates the Lung-Left, green the Lung-Right, red the GTV-1 region, and black the Spinal-Cord. There is no GTV-2 image of the patient in the 28th CT image for LUNG1-243.

It is not possible to directly access the available segmented images. To access segmented images, the Radiotherapy Structure Set (RTSTRUCT) document in each patient’s folder is used. Since the segmented image information of patient number LUNG1-128 was not available in the dataset used in this study, data from 421 patients were used. The DICOM format file located in the RTSTRUCT folder contains many meta-data. A description of the meta-data used within the scope of the study is given in Table 4.

**Figure 4:**
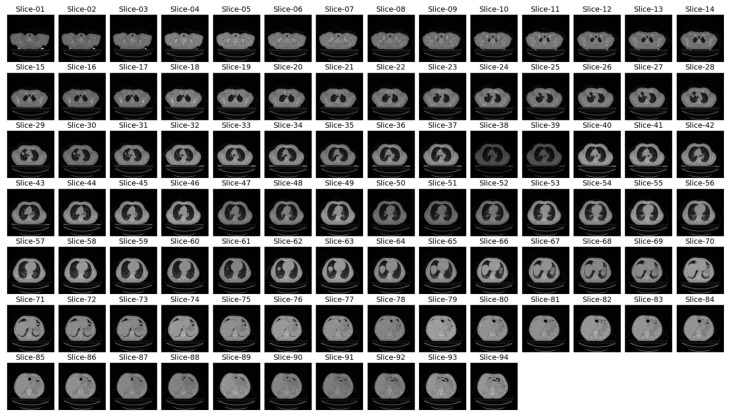
CT slices from patient LUNG1-243 and representation of tumor areas with GTV-1 labeling.

In the previous literature addressing similar problems to ours, only tumor regions from patients labeled as GTV-1 were utilized. The dataset contains a variable number of images with GTV-1 labeled tumors, ranging from 2 to 97. Figure 4 shows CT slices from a patient (LUNG1-243) with GTV-1 regions marked. There are 94 slices and 13 GTV-1 labeled tumor regions (Slice 21 through Slice 33) for this patient. For all patients in the dataset, Slice Thickness is given as 3.0 mm and Pixel Spacing is 0.977 mm.

**Figure 5:**
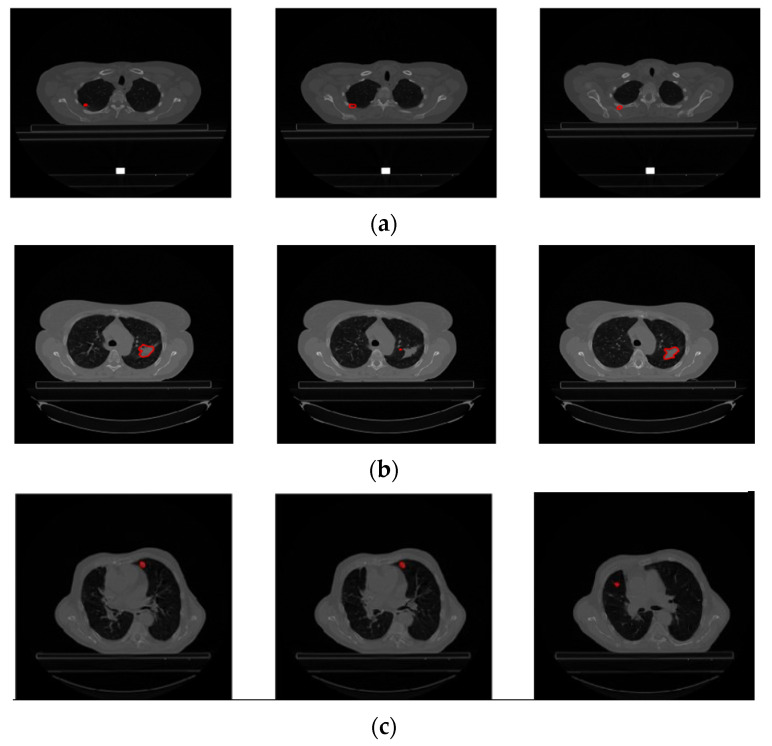
Incorrectly segmented images. (**a**) Incorrect labeling of tumor regions (LUNG1-158), (**b**) interpolation of segmentation images in consecutive slices (LUNG1-127), (**c**) presence of more than one tumor in an image (LUNG1-326).

Upon examination of Figure 4, it is evident that GTV-1 information is absent in each slice. As a result of a detailed analysis of the *Lung1* dataset, Braghetto stated that 5 patients were incorrectly segmented due to incorrect labeling of tumor regions, 62 patients due to interpolation of segmentation images in consecutive slices, and 3 patients due to the presence of more than one tumor in one image [23]. The representation of each error type is given in Figure 5.

### 2.2. Preprocessing Image Data

In order to appropriately utilize the acquired data in the models, it is necessary to perform preprocessing steps initially. These preprocessing steps are presented in the following two subsections as processing of DICOM images and rectification of errors in the database.

#### 2.2.1. Processing of DICOM Images and RTSTRUCT Data

Within the scope of this study, the initial task involves locating slices containing GTV-1 regions. This process was conducted by following the steps outlined in Braghetto’s study [23], thereby benefiting from their insights.

1. By reading the RTSTRUCT file of each patient, the index of the segmentation image with the GTV-1 label in the file is found (the “*ROIName*” property of each label ID in the “*StructureSetROISequence*” is looked at and the index of the region with the “*GTV-1*” label is kept).
2. Using the index information, the number of slices containing cancerous cells (labeled GTV-1) in the patient can be found (“*ContourSequence*” information belonging to the GTV-1 index is used in the “*ROIContourSequence*”).
3. The ID information of the segmentation image with the GTV-1 label is obtained (“*ReferencedSOPInstanceUID*” information of the 0th element of the “*ContourImageSequence*” is used for the segmentation image containing each cancerous area).
4. The ID information of the relevant patient’s CT slices is obtained (“*SOPInstanceUID*” information is used).
5. The ID information of patient CT slices and the ID information of slices labeled GTV-1 have common elements. In this way, information about the slices containing GTV-1 belonging to the patient is obtained.

The region of interest (RoI) is obtained by using the coordinate information of the tumor area in the slices labeled GTV-1. The following steps were taken to perform this task.

1. Borders in each segmentation image with the GTV-1 label were found (with the help of “*ContourData*” information). “*ContourData*” expresses the information of the tumor regions in mm for the x, y, and z axes (For example, ‘−56.15’, ‘−230.73’, ‘−491.5’).
2. The border of the tumor area obtained in mm is converted into pixels.
  - Each image in CT slices labeled GTV-1 has a reference point in the x, y, and z axes (with the help of “*ImagePositionPatient*”).
  - The pixel spacing of each image in the CT slices labeled GTV-1 in the x, y, and z axes is found (with the help of “*PixelSpacing*”).
  - Coordinate transformation is performed using Equation (1). In the equation, $x_{mm}$ and $y_{mm}$ represent the coordinate information in millimeters, $x_{s}$ and $y_{s}$ represent the pixel spacing of the image, $x_{0}$ and $y_{0}$ represent the position of the image reference frame, and $x_{pixel}$ and $y_{pixel}$ represent the position of the image in pixels.
    (1) $$x_{pixel}=\frac{x_{mm}-x_{0}}{x_{s}}\;and\;y_{pixel}=\frac{y_{mm}-y_{0}}{y_{s}},$$

Then, DICOM slices were converted to JPEG format for cropping tumor regions and performing other preprocessing. While the conversion process was performed, the values in the DICOM images were normalized between 0–255. Equation (2) was used in the normalization process.

(2) $$jpeg=round\left(\frac{dcm-\min\left(dcm\right)}{\max\left(dcm\right)}\times 255\right)$$

In the above equation, dcm and jpeg expressions refer to DICOM and JPEG pixel values, respectively.

#### 2.2.2. Handling Incorrectly Segmented Images in the Dataset

**Figure 6:**
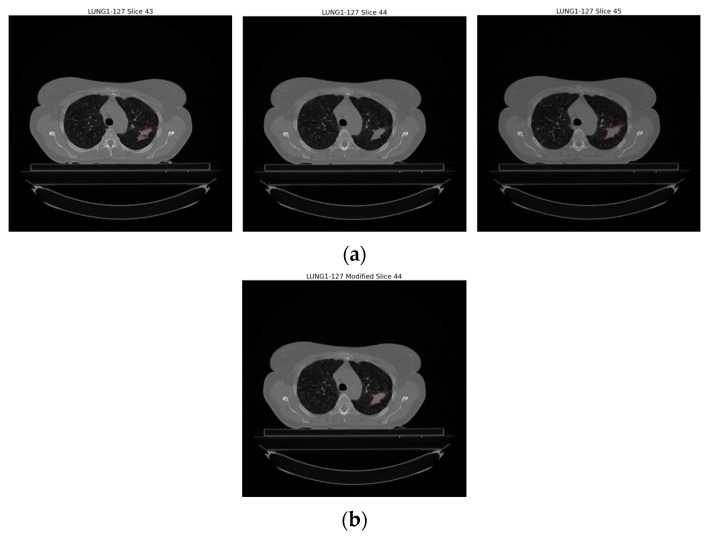
Illustration of the incorrectly segmented slice caused by interpolation in sequential segmentation in slices of an example patient (LUNG1-127). (**a**) Original version of Slice 43, Slice 44, Slice 45, (**b**) interpolation of Slice 44 based on Slice 43 and Slice 45.

Improper images in the dataset given in the previous section were re-examined and solutions were developed against these incorrectly segmented images. As a result of the analysis, no solution was developed in Figure 5a because it was not possible to verify the error type by the clinician. Against the error type in Figure 5b, when there were sudden changes in the number of pixels covering the regions with each GTV-1 label, the relevant error was detected and the GTV in the slice where the error was found was made by interpolating between the slice before and the slice after where the errors were found. Figure 6 shows the update in the slice where the error was found. Against the error type in Figure 5c, it was determined whether the tumor was on the right or left, and only one region was focused on.

#### 2.2.3. Ready-to-Use Input Images for the Model

**Figure 7:**
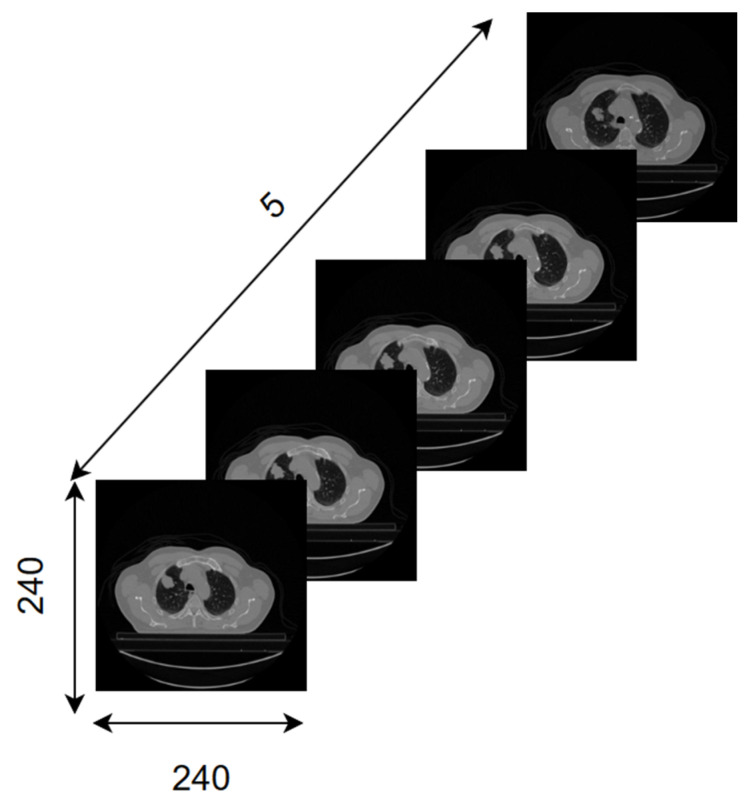
An example input image for the model.

While performing the tests, the censored observations are discarded, and the input images of the remaining patients are converted to gray level, 240 × 240 in size, and 5-slice (240 × 240 × 5). When adjusting the 5-slice images, the slice with the largest tumor area among the slices containing the tumor areas is selected. In order to perform this process, first, the contours of the tumor regions are determined in the CT slices of each patient containing GTV-1. Using the extreme points of the tumor perimeter, the area is found by drawing the minimum rectangle surrounding the tumor area. To preserve spatial information, the largest tumor slice has two adjacent slices before and two after that contain the tumor. If the number of slices containing the tumor region is greater than or equal to 5, it and four neighboring slices are kept. If the number of slices containing tumor regions is less than 5, they are oversampled (copied) and saved until the number of slices containing tumors is five. Finally, all input images are normalized between 0–1 to ensure that the neural network gives more successful results. Figure 7 shows an example input image sent to the model.

### 2.3. 3D ResNet-34 Architecture

In this study, 2-year survival classification was performed using only CT images with the 3D ResNet-34 network. ResNet architectures are deep neural network architectures that add extra shortcut connections to the model and vary between 18 and 152 layers in order to eliminate the vanishing (zero) or exploding (large value) gradient problems caused by the increasing number of layers in deep convolutional neural networks [32]. In the tests performed, the 5-slice 3D input images were sent to the 3D version of the ResNet-34 model.

### 2.4. A New Feature for Clinical Data: GTV1-SliceNum

Tumor thickness is the measurement in millimeters of the perpendicular distance between the highest point of the tumor surface and the deepest point of the infiltrative front of the tumor [33]. There are many studies in the literature that reveal a significant relationship between tumor thickness and overall survival. In [34], it was noted that the median survival time was 24.2 months for a lung pleural thickness of less than or equal to 5.1 mm, and 17.7 months for a thickness exceeding this value. Hsu et al. divided NSCLC patients into three groups, taking into account operation notes (ONs) and pathology reports (PRs), and performed a 5-year survival analysis. According to the ONs and PRs, the survival results were 70.1% for Group 1 patients with tumors 3 cm or smaller (ON and PR); 49.1% for Group 2 patients with tumors larger than 3 cm (ON and PR); 51.1% for Group 3 patients with tumors larger than 3 cm (ON) and tumors 3 cm or smaller (PR) [35]. Gonzalez-Moles et al. showed that tumor thickness in tongue cancer has the greatest impact on survival, and patients with a tumor thickness of less than or equal to 3 mm had a 5-year survival of 85.7%; 58.3% in patients with tumor thickness between 4–7 mm; and 57% in patients with >7 mm [36]. In the study, it was emphasized that tumor thickness was significantly associated with survival in Merkel Cell Carcinoma (MCC). The 5-year disease-free survival was found to be 18% in tumors >10 mm thick and 69% in tumors ≤ 10 mm thick, and the disease-specific 5-year survival was found to be 74% in tumors >10 mm thick and 97% in tumors ≤ 10 mm thick [37].

Slice Thickness for CT image slices in the dataset is specified as 3.0 mm. Each patient has a variable number of slices (75–297) as well as a variable number of GTV-1 labeled slices (2–97). Therefore, the number of slices with different numbers of GTV-1 tags in the dataset may constitute a meaningful feature for survival classification. For example, while one patient has only 2 slices with the GTV-1 label, another patient has 21 slices with the GTV-1 label, which, in a sense, indicates the tumor thickness. For each patient in the dataset, the region with the GTV-1 label in each slice is obtained through the RTSTRUCT label called *ROIName*, and the total number of slices with the GTV-1 label for each patient is added to the clinical data as a new feature.

**Figure 8:**
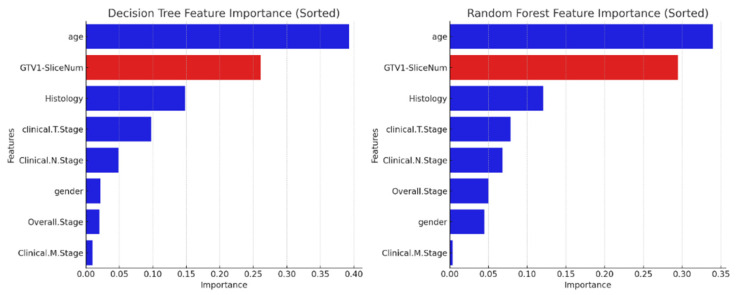
Importance score of features in classification of GTV1-SliceNum feature for *Lung1* dataset. (**left**) Decision Tree. (**right**) Random Forest.

Feature importance score is a value that measures the contribution of each feature (or variable) in a machine learning model to its predictive performance. The calculated feature importance score provides detailed information about the dataset and reveals which feature(s) is/are more dominant in the relevant problems. In this way, the features with high scores can be selected, while the features with low scores can be eliminated and the model can be simplified. Statistical correlation scores, coefficients calculated of models, and many other techniques are used when calculating feature importance scores. The importance score of the added *GTV1-SliceNum* feature was tested with a Decision Tree and a Random Forest, which are well-known machine learning methods, and the results are shown in Figure 8.

As depicted in Figure 8, the proposed *GTV1-SliceNum* feature has demonstrated its significance by ranking as the second most influential feature in survival classification. The proposed *GTV1-SliceNum* feature can be considered a special interpretation of a popular GTV concept that is widely used in oncology as a prognostic factor. The GTV measure had been mentioned as significant in a pivotal study, presented in [38], which involved stage III NSCLC patients and demonstrated its critical role in survival prediction and treatment planning.

### 2.5. A New Loss Function: Penalized Binary Cross Entropy (PEN-BCE)

During training of neural networks, the loss function is very important to learn model parameters and produce robust results. Loss functions are handled in different ways for classification and regression problems. A cross-entropy loss function, which is a measure of the difference between real class labels and the probabilities predicted by the model, is often preferred in classification.

The performance of the model’s predictions between two classes is measured by using the binary cross-entropy loss function, which is specialized for binary classification problems. However, this loss function does not directly account for false positives (FPs) and false negatives (FNs), which provide critical information about how the model performs in real-world scenarios. However, to better adapt to real-world scenarios and classify imbalanced datasets, different loss functions can be used, such as weighted cross-entropy loss, focal loss (FL) [39], asymmetric loss (ASL) [40], and real-world weight cross-entropy loss (RWWCE) [41].

The loss functions mentioned above do not fully address the FN and FP cases. The proposed *Penalized Binary Cross Entropy Loss (PEN-BCE)* provides a loss function that is more suitable for real-world scenarios by adding deviations in the produced output probability values as a penalty parameter for both FN and FP cases. To understand the PEN-BCE loss function, the binary cross-entropy loss given in Equation (3) should first be examined.

(3) $$BCE=-\frac{1}{N}\sum_{i=1}^{N}y_{i}\log\left(p_{i}\right)+\left(1-y_{i}\right)\log\left(1-p_{i}\right),$$

In this loss function, *N* refers to the total number of training data, *y*_i_ refers to the ground truth target variable of the relevant training data, and *p_i_* refers to the classification probability of the relevant training data. The weighted *BCE* loss used with reference to Equation (3) includes an additional weight (*w*) parameter that emphasizes the importance of positive labels, as in Equation (4).

(4) $$BCE_{weighted}=-\frac{1}{N}\sum_{i=1}^{N}{wy}_{i}\log\left(p_{i}\right)+\left(1-y_{i}\right)\log\left(1-p_{i}\right),$$

Unlike the *BCE_weighted_* loss, the focal loss has been reshaped to reduce the weight of easily classified examples for problems arising from imbalanced datasets, thus enabling training to focus on difficult examples [39]. To achieve this, a modulation factor ${\left(1-p_{t_{i}}\right)}^{\gamma }$ was added to the cross-entropy loss with the focusing parameter γ (γ ≥ 0), as given in Equation (5).

(5) $$FL=-\frac{1}{N}\sum_{i=1}^{N}y_{i}\log\left({p_{t}}_{i}\right)\;{\left(1-p_{t_{i}}\right)}^{\gamma },$$

Here, *p_ti_* varies depending on the value of the label of the relevant training example (*p_i_* if the label, *y_i_*, is 1, otherwise 1 − *p_i_*).

Ridnik et al., in their study, proposed a new loss function that allows dynamically reducing the weight of easy negative examples and exceeding difficult thresholds [40]. The ASL loss function detailed in Equation (6) combines the mechanisms of asymmetric focusing and probability shifting.

(6) $$ASL=-\frac{1}{N}\sum_{i=1}^{N}y_{i}\log\left(p_{i}\right)\;{\left(1-p_{i}\right)}^{\gamma_{+}}+\left(1-y_{i}\right)\log\left(1-{p_{m}}_{i}\right){\left({p_{m}}_{i}\right)}^{\gamma_{-}},$$

The γ+ and γ- parameters given in Equation (6) are the focusing parameters that adjust the focusing levels of positive and negative samples. Asymmetric focusing reduces the contribution of negative samples to loss when their probabilities are low and adds a probability shift parameter ((${p_{m}}_{i}=max(p_{i}-margin,\;0)$)), which is an additional mechanism that performs hard thresholding of easy negative samples, that is, completely eliminates negative samples when their probabilities are very low to the loss function. The margin value specified in the function is greater than 0 and is only integrated into the right side of the equation to obtain asymmetric probability shifting focus loss.

Ho and Wookey define the RWWCE loss function, and the weights related to the cost of missing positive and negative samples separately, as given in Equation (7) [41].

(7) $$RWWCE=-\frac{1}{N}\sum_{i=1}^{N}{w_{mcfn}y}_{i}\log\left(p_{i}\right)+w_{mcfp}\left(1-y_{i}\right)\log\left(1-p_{i}\right),$$

While *w_mcfn_*, given in Equation (7), expresses the marginal cost of the false negative relative to the true positive, *w_mcfp_* expresses the marginal cost of the false positive relative to the true negative.

The loss functions given above are derived from BCE-based loss functions for real-world scenarios. However, these functions do not directly address or penalize the possibility of incorrect predictions. Therefore, the proposed PEN-BCE loss function takes advantage of the loss functions in the literature to both penalize misclassifications caused by FPs and FNs and emphasize the effect of incorrectly estimated probabilities. In order to achieve this, PEN-BCE adds a penalty parameter to the BCE loss, as seen in Equation (8).

(8) $$\begin{array}{c} PEN-BCE=-\frac{1}{N}\sum_{i=1}^{N}y_{i}\log\left(p_{i}\right)+\left(1-y_{i}\right)\log\left(1-p_{i}\right)+\propto y_{i}\max(0,\;{p_{i}}_{FN}-\\ {p_{i})}^{2}+\beta \left(1-y_{i}\right){\max\left(0,\;p_{i}-{p_{i}}_{FP}\right)}^{2}, \end{array}$$

**Figure 9:**
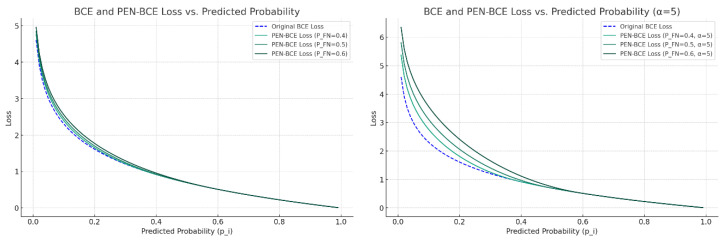
Change of BCE and PEN-BCE loss functions according to the estimated probability. (**Left**) for ∝ = 1 and (**right**) for ∝ = 5.

∝ and *β* given in Equation (8) represent FN and FP weights, respectively, and *p_iFN_* and *p_iFP_* parameters refer to FN and FP probability threshold values, respectively. In addition to the standard Binary Cross Entropy Loss function, the function includes extra terms for the FN and FP cases. Thanks to these additional terms, the model can impose more penalties on FNs and FPs. For *y_i_* = 1 value of the function, the equation is simplified as $PEN-BCE=-\log p_{i}+\propto \;.{max(0,\;{p_{i}}_{FN}-p_{i})}^{2}$. Using this equation, PEN-BCE loss values for a range where *p_i_* values vary between 0 and 1 can be plotted, as shown in Figure 9.

The original Binary Cross Entropy Loss (blue dashed lines) function and PEN-BCE Loss function (for different *p_FN_* values) are given above. PEN-BCE produces higher loss values compared to the original BCE function, especially at lower prediction probabilities (when *p_i_* values are low). This indicates that the model aims to reduce FN predictions by giving them a larger penalty. Increasing the value of *p_FN_* means that the penalty will become larger, and the model will be more directed towards minimizing false negatives. Additionally, increasing the value of ∝ results in more penalties for underestimation probabilities. This encourages the model to further reduce such errors by penalizing false negative predictions more stringently. Looking at the graph, it can be seen that, as the value of ∝ increases, the loss values increase greatly, especially for low probabilities.

## 3. Results

In this study, we conducted a 2-year survival classification analysis using the 3D ResNet-34 architecture on CT images of lung cancer patients, employing the *NSCLC-Radiomics (Lung1)* dataset. Subsequently, various ablation studies were conducted to assess their impact on classification efficacy, followed by a detailed analysis presentation.

All experiments detailed in the subsequent sections were conducted on a computing system equipped with Pop!_OS 22.04 LTS, powered by an AMD Ryzen 9 5980HS with Radeon Graphics CPU @ 3.30GHz, 32 GB LPDDR4X RAM, and an NVIDIA GeForce^®^ RTX3080 eGPU. The system utilized CUDA 11.2 and CUDNN 8.1, operating within the Keras framework with a Tensor-flow 2.8.0 backbone.

During the experiments, the dataset was split into training and testing sets using an 85%–15% ratio. The *Lung1* database utilized in this study comprises clinical data and CT images from 422 patients. However, due to errors in the segmentation file for patient LUNG1-128, the clinical data and CT slices of this patient were excluded from the experiment. Therefore, tests were conducted on the remaining 421 patients. For those 421 observations, there were 122 patients (32.7%) with a survival time exceeding 2 years, while 251 patients (67.3%) had a survival time of less than 2 years at the 2-year classification threshold. Maintaining class balance during the train–test split was prioritized. Hence, the 2-year classification threshold in the randomly generated training set was adjusted to 30.9% and 69.1% for survival times exceeding 2 years and those below 2 years, respectively, mirroring the proportions observed in the entire dataset.

### 3.1. Two-Year Survival Classification with 3D ResNet-34 Model

In model training, *sigmoid* was defined as the activation function in the output layer, and binary cross entropy was defined as the loss function. A 5-fold cross-validation process was performed in training the model. The optimization method used when training the models was Stochastic Gradient Descent (SGD), and the initial learning rate was set to 2 × 10^−5^ and the weight decay parameter was set to 1 × 10^−6^. To ensure better convergence of SGD, a Nesterov accelerator was used, and the momentum value was determined as 0.9. In training the models, the batch size was set to 16, and, if the validation loss remained constant for 25 cycles, the learning rate was reduced by 0.9. Additionally, Early Stopping was applied to prevent overlearning of the model. Accuracy (ACC) and Area Under the Curve (AUC) metrics were used to evaluate the success of the models and the model was trained for 200 epochs.

**Figure 10:**
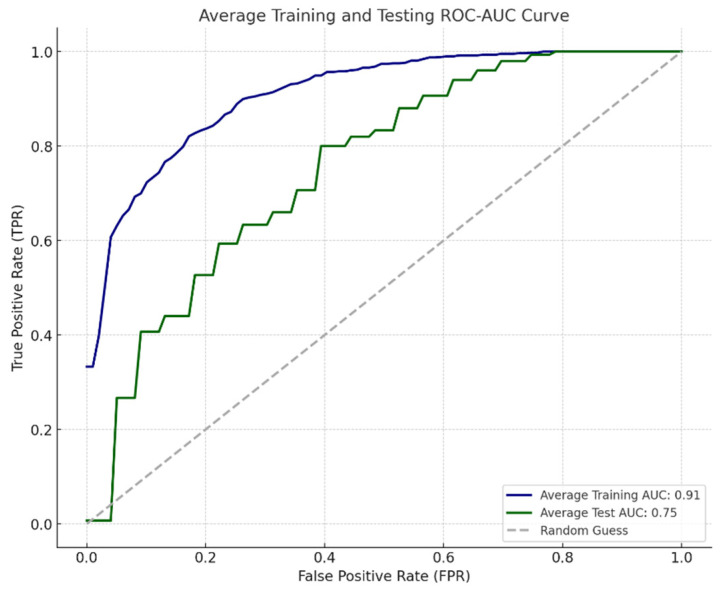
Two-year survival classification results (ROC-AUC) with 3D ResNet-34 model.

The conducted tests involved the exclusion of censored observations, with 5-slice 3D input images being fed into the ResNet-34 model. The resultant tests yielded an average test loss value of 0.6380, with average test accuracy (ACC) and area under the receiver operating characteristic curve (AUC) values obtained as 0.6377 and 0.7548, respectively. Furthermore, the ROC-AUC curve for the test is presented in Figure 10. 

### 3.2. Ablation Study

In this study, an ablation study was conducted to comprehend the influences of slice numbers in input images, data augmentation, censored observations, and the proposed loss function on resolving the survival classification problem under consideration. The contributions of each individual component to the achievement are elucidated in subsequent paragraphs.

Effect of Number of Slices: During the tests, the impact of varying the number of slices in the input image transmitted to the 3D CNN architecture on the classification outcome was demonstrated. In this context, the efficacy of the 5-slice structure utilized in the experiment conducted in the previous subsection was compared to that of the 4-slice structure, and the findings are presented in Table 5.

Effect of Data Augmentation: While performing the tests, data augmentation was performed by rotating the input images and shifting them horizontally and vertically. The comparison of the data-augmented test and the test performed in Section 3.1 is given in Table 6.

Effect of Censored Observations: The *Lung1* dataset contains 11.4% censored observations. Katzman et al. stated that, when survival analysis is considered as a standard regression problem, right-censored data should be discarded [42]. Right-censoring is a statistical method used to estimate the time until an event by taking into account the time elapsed from the moment an event is observed. It is often employed in survival analysis, where the survival time of a group of individuals regarding a specific event (such as death) is examined. However, some individuals may not experience the event during the observation period or fail to report their outcomes. Such instances are termed right censoring because the dates of the event occurrence are censored from the right side (i.e., beyond the end of the observation period). Since survival analysis was considered a survival classification problem in this study, extra tests were performed by adding censored data. In tests performed with censored observations, if the patient’s follow-up exceeded 730 days (2 years), the patient’s survival class was set to more than 2 years. To observe the effect of censored observations, the classification performance in cases without censored observations (373 patients) and in cases with censored observations (421 patients) was compared and shown in Table 7.

**Figure 11:**
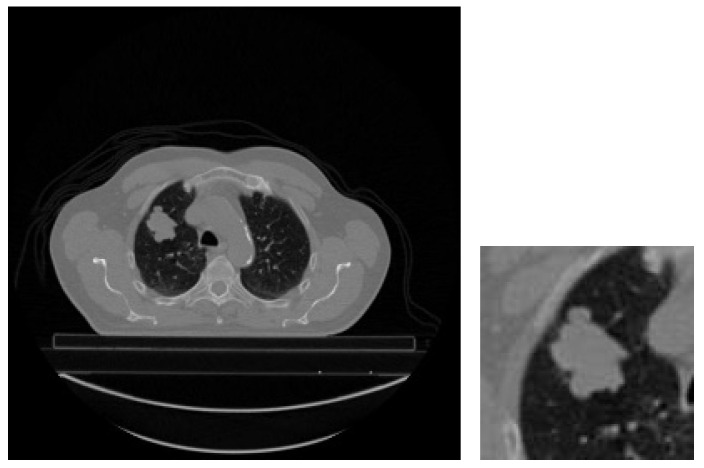
Original slice and cropped image containing the largest GTV-1 tumor (LUNG1-243).

Effect of the Input Image: In the experiments, the main motivation was to facilitate a more efficient process by utilizing both the original versions of CT slices belonging to patients and cropped regions of interest (ROIs) corresponding to GTV-1 tumor areas. Within this scope, the initial step involves cropping the tumor regions from the images. This necessitates identifying the surroundings of the tumor regions within the CT slices containing GTV-1 tumors. Subsequently, the midpoint of the minimum rectangle surrounding the tumor region is determined using the endpoints of the tumor boundary. Finally, the tumor region is cropped to a size of 128 × 128 pixels with the midpoint of the rectangle as the center. Figure 11 illustrates the cropped tumor region alongside the CT slice containing the largest GTV-1 circumference for a patient (LUNG1-243).

To assess the impact of the input image, 5-slice input images comprising the GTV-1 RoI regions are provided to the model. The classification results are contrasted with those obtained in the experiment conducted in the previous subsection and presented in Table 8.

Impact of Loss Function: While the binary cross-entropy loss function is commonly employed for binary classification tasks, it may prove inadequate for directly addressing real-world scenarios and imbalanced datasets as it does not explicitly consider false positives and false negatives produced by the model. To address this limitation, the proposed *PEN-BCE* loss function incorporates penalty parameters to specifically address FP and FN occurrences alongside the BCE loss. Consequently, the influence of the proposed loss function on classification success was evaluated by comparing it with the test conducted in the previous subsection, and the outcomes are presented in Table 9.

**Figure 12:**
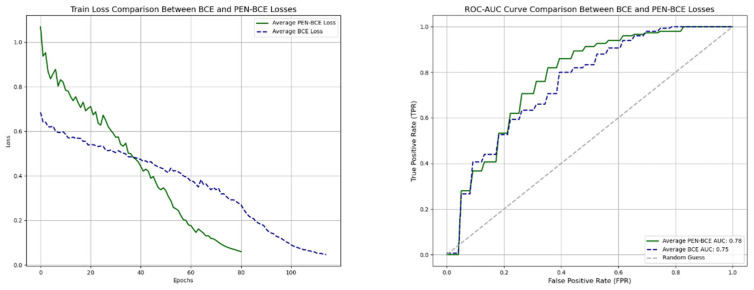
Comparison between BCE and PEN-BCE. (**Left**) Test loss; (**Right**) Test ROC-AUC.

Figure 12 illustrates the comparisons between PEN-BCE and BCE results in terms of loss and ROC-AUC curves. 

Impact of Parameters in the Loss Function: The PEN-BCE loss function includes four new hyper-parameters (∝, *β*, *p_FN_*, *p_FP_*) in addition to the existing hyper-parameters in the training process. Among these hyper-parameters, ∝ and β represent FN and FP weights, respectively, and p_FN_ and p_FP_ parameters refer to FN and FP probability threshold values, respectively. Within the scope of this study, tests were carried out on the *Lung1* dataset with various hyper-parameter combinations, as given in Table 10. Hyper-parameter combinations were selected empirically.

As indicated in Table 10, the hyperparameter configuration yielding the highest accuracy (ACC) and area under the curve (AUC) success was attained with the following values: ∝ = 1.0, *β* = 5.0, *p_FN_* = 0.50, and *p_FP_* = 0.20.

## 4. Discussion

The study’s findings, resulting from a range of tests conducted, are presented below.

1. The newly introduced feature (*GTV1-SliceNum*) holds significant importance in survival classification, as evidenced by its correlation with the number of tumor slices and survival duration, akin to the relationship observed between tumor thickness and overall survival. Moreover, it has been noted that the quantity of slices forwarded to the 3D ResNet-34 model influences the success of classification.
2. Notably, superior outcomes are achieved upon discarding censored data. This observation aligns with the assertion made by Katzman et al. in their study [42], suggesting that right-censored data should be excluded when treating survival problems as standard regression tasks.
3. It has been noted that classification performance tends to decrease when utilizing solely the GTV-1 tumor regions, designated as regions of interest, within the input image. This phenomenon may stem from the fact that, beyond the tumor itself, surrounding tissues or other structures relevant to the tumor could also bear significance in survival prediction. Moreover, while convolutional neural networks (CNNs) excel at automatically extracting features from input images, training the model solely on a restricted region might hinder its ability to grasp broader patterns comprehensively.
4. The observation reveals that the AUC metric yields more reliable results compared to the accuracy metric. This phenomenon is attributed to the AUC metric’s superior performance in imbalanced datasets, as it effectively mitigates the shortcomings of accuracy. Specifically, accuracy metrics can inaccurately depict model performance by favoring the larger class, even if the model’s predictive ability for the smaller class is poor.
5. It has been noted that the proposed novel loss function (*PEN-BCE*) enhances classification performance and adeptly manages false positive (FP) and false negative (FN) cases.
6. In the conducted tests, it was evident that the proposed method for the *Lung1* dataset outperformed all previous studies documented in the literature. The comparative analysis of the conducted tests with the studies listed in Table 1 is presented in Table 11.

While our study provides valuable insights into the field of survival classification, it is not without limitations. The reliance on a single dataset and the inherent complexities of medical image analysis pose challenges that warrant further exploration in future research endeavors. In conclusion, this study contributes to a deeper understanding of survival classification in lung cancer patients and offers practical implications for clinical decision-making. By addressing the identified gaps and leveraging innovative methodologies, future research can continue to advance the field toward more accurate and personalized prognostic models.

## 5. Conclusions

In this study, we aimed to address various aspects of survival classification in lung cancer patients using advanced image analysis techniques and novel methodologies. Through a comprehensive analysis of the *Lung1* dataset, several key findings emerged. The primary conclusion of our study is that the integration of imaging features and a novel loss function significantly improves the performance of survival predictions for lung cancer patients. Our investigation revealed the importance of incorporating detailed features such as the number of tumor slices and the utilization of surrounding tissues in the input image for improved classification accuracy. This finding shows a similar dynamic to the relationship between tumor thickness and overall survival and emphasizes how critical a detailed examination of tumor structure is in survival predictions. In the clinic, tumor sizes are usually assessed by systems such as TNM staging, but the use of the new quantitative feature revealed by our study may contribute to the development of more accurate and personalized prognostic models. The 3D CNN architecture used in this study can automatically extract a wide range of features from the visual CT images. These features can capture complex patterns, textures, and spatial relationships within the tumor and surrounding tissues. Furthermore, the effectiveness of the proposed *PEN-BCE* loss function in handling false positive and false negative cases was demonstrated, leading to enhanced classification performance. This is of great importance in terms of improving model performance, especially considering that misdiagnoses can have serious consequences in the medical field. Notably, our results surpassed those reported in previous studies, underscoring the significance of our approach in advancing the state-of-the-art in survival classification for lung cancer and demonstrating the potential of AI-based approaches, providing an important basis for future research in this field. The predictive models and post-treatment monitoring pathways used in current clinical practice are generally based on standard clinical parameters and imaging techniques. This study demonstrates how effective image analysis and innovative methodologies can be in clinical applications and can make significant contributions to the development of clinical decision support systems and the creation of more personalized treatment strategies.

For future research, testing the model on larger and more diverse datasets will increase the generalizability of the findings. Moreover, applying similar methodologies to different tumor types and other types of cancer could expand the overall performance and scope of the application of the model. In addition, the development of more integrated and comprehensive models for post-treatment monitoring pathways and long-term follow-up of patients may provide more accurate and reliable results in survival analysis.

## Figures and Tables

**Table 1:** Summary of the literature studies addressing the survival classification problem for lung cancer.

Ref.	Dataset	Model (Best)	Classification Type	Performance (Best)
[15]	SEER	ANN	3-class Class 1: ≤6 months, Class 2: 6–24 months, Class 3: >24 months	ACC: 0.7118
[16]	NCBI-GEO	Bimodal DNN (proposed)	2-class 5-year cut-off	ACC: 0.7544 AUC: 0.8163
[17]	TCGA-LUSC	CapSurv (proposed)	2-class 1-year cut-off	AUC: 0.702
[18]	Moffitt Cancer Center	Nearest Neighbor	2-class 2-year cut-off	ACC: 0.825
[19]	NLST	MS-ResNet (proposed)	3-class Class 1: ≤36 months, Class 2: 36–60 months, Class 3: >60 months	ACC: 0.8678
[20]	Henan Provincial People’s Hospital	Residual CAE (proposed)	2-class 1-year cut-off	ACC: 0.75 AUC: 0.71
[21]	Lung1 and Lung2	Random Forest	2-class 2-year cut-off	AUC: 0.66
[22]	Lung1	Logistic Regression	2-class 2-year cut-off	AUC: 0.699
[24]	Lung1	Random Forest	2-class 2-year cut-off	AUC: 0.67

**Table 2:** Clinical data and descriptions in the dataset.

Clinical Data	Description
PatientID	Represents the patient’s identification (LUNG1-XXX).
age	Represents the patient’s age in days.
clinical.T.Stage	Represents the patient’s tumor stage. Indicates the size of the tumor and where it is located.
Clinical.N.Stage	Represents the patient’s lymph node stage. Indicates whether the tumor has spread to the lymph nodes.
Clinical.M.Stage	Represents the patient’s metastasis stage. Indicates whether the cancer has spread to other parts of the body.
Overall.Stage	By combining the patient’s T, N, M results, the stage of cancer is determined.
Histology	Indicates in which tissue (large cell, squamous cell carcinoma, etc.) the cancer is available.
gender	Represents the patient’s gender.
Survival.time	Represents the time until an event of interest occurs for each patient in days from the start of treatment.
deadstatus.event	Indicates that the patient died due to cancer or that the observation could not be completed due to censorship.

**Table 3:** DICOM tags and descriptions used.

Meta Data	Description
SOP Instance UID	Represents the identification for each slice.
Pixel Array	Represents the 512 × 512-pixel matrix of the image data.
Slice Position	Represents the z-coordinates of the slices along the axial axis.
Rescale Intercept	Intercept parameter used to transform the pixel matrix.
Rescale Slope	Slope parameter used to transform the pixel matrix.
Slice Thickness	Represents the distance between two consecutive slices in mm.
Pixel Spacing	Represents the distance between pixels in the Pixel Array component.

**Table 4:** RTSTRUCT tags and descriptions used.

Meta Data	Description
Referenced SOP Instance UID	Represents the identity of the slice on which the segmentation process is applied.
Structure Set ROI Sequence	Contains ROI information for the current structure set.
ROI Contour Sequence	Refers to the boundary sequences that will define the ROI.
Contour Sequence	Refers to boundary sequences.
Contour Image Sequence	Contains arrays of images containing the boundary.
ROI Name	It is the array containing the names of the segmentation sets for slices.
Contour Data	These are the values that hold the boundary data of segmented regions.

**Table 5:** Effect of number of slices utilized.

5-Slice Input Image (Original Test)			4-Slice Input Image		
Test Loss	Test ACC	Test AUC	Test Loss	Test ACC	Test AUC
0.6380	0.6377	0.7548	0.8484	0.6415	0.6230

**Table 6:** Effect of data augmentation.

Without Data Augmentation (Original Test)			With Data Augmentation		
Test Loss	Test ACC	Test AUC	Test Loss	Test ACC	Test AUC
0.6380	0.6377	0.7548	0.6535	0.6025	0.7563

**Table 7:** Effect of censored data.

Without Censored Data (Original Test)			With Censored Data		
Test Loss	Test ACC	Test AUC	Test Loss	Test ACC	Test AUC
0.6380	0.6377	0.7548	0.7054	0.6000	0.6419

**Table 8:** Effect of input image.

Original Slice Including GTV-1 (Original Test)			GTV-1 ROI		
Test Loss	Test ACC	Test AUC	Test Loss	Test ACC	Test AUC
0.6380	0.6377	0.7548	0.7637	0.6113	0.6291

**Table 9:** Impact of loss function.

Binary Cross Entropy (BCE) Loss (Original Test)			Penalized Binary CE Loss (PEN-BCE) $\left(\propto =1.0,\;\beta =5.0,\;p_{FN}=0.5,\;p_{FP}=0.2\right)$		
Test Loss	Test ACC	Test AUC	Test Loss	Test ACC	Test AUC
0.6380	0.6377	0.7548	0.8131	0.7434	0.7768

**Table 10:** Classification performance of some PEN-BCE hyper-parameters.

∝	*β*	*p_FN_*	*p_FP_*	Test Loss	Test ACC	Test AUC
5.00	1.00	0.40	0.50	0.6933	0.6755	0.7629
5.00	1.00	0.60	0.50	0.6691	0.6792	0.7532
5.00	1.00	0.70	0.50	0.7114	0.6151	0.7428
1.00	5.00	0.50	0.20	0.8131	0.7434	0.7768
1.00	5.00	0.50	0.30	0.8287	0.6302	0.7117
1.00	5.00	0.50	0.40	0.7692	0.7019	0.7265
5.00	5.00	0.50	0.50	0.6631	0.6415	0.7752
5.00	5.00	0.75	0.25	1.0310	0.6830	0.7525
5.00	5.00	0.60	0.20	0.9849	0.7094	0.7439

**Table 11:** Comparison of the proposed methods that use classification in a similar manner to our study by using the *Lung1* dataset.

Ref.	Model (Best)	Classification Type	ACC	AUC
[21]	Random Forest	2-class 2-year cut-off	-	0.66
[22]	Logistic Regression	2-class 2-year cut-off	-	0.699
[24]	Random Forest	2-class 2-year cut-off	-	0.67
Proposed	5-sliced 3D ResNet-34 w/BCE Loss	2-class 2-year cut-off	0.6377	0.7548
Proposed	5-sliced 3D ResNet-34 w/PEN-BCE Loss	2-class 2-year cut-off	0.7434	0.7768

## Data Availability

The data that support the findings of this study are openly available in the NSCLC-Radiomics cancer imaging archive at https://doi.org/10.7937/K9/TCIA.2015.PF0M9REI, reference number [28].
